# Supplementary material for: Mental health and health behaviours before and during the initial phase of the COVID-19 lockdown: longitudinal analyses of the UK Household Longitudinal Study
Source: J Epidemiol Community Health. 2020 Sep 24;75(3):224–31. doi: 10.1136/jech-2020-215060 (PMC7892383; doi:10.1136/jech-2020-215060)
Supplement: Supplementary data [file jech-2020-215060supp001.pdf]

Appendix 1

Figures and Tables

Figure S1: STROBE flowcharts for cross-sectional and longitudinal analyses *3*

Box S1: Lockdown measures introduced in four nations of UK, March-April 2020 .... *4*

Table S1: Comparison of Wave 9, COVID-19 (CA) Wave and Longitudinal Sample *6*
Table S2: Mental health and health behaviours before and during the COVID-19 lockdown *8*
Table S3: Psychological distress (GHQ-12, 4+ cut-off) and the interactions between period (pre-COVID-19 and during COVID-19) and age group, gender, ethnicity and education level *10*
Table S4: Loneliness and the interactions between period (pre-COVID-19 and during COVID-19) and age group, gender, ethnicity and education level *12*
Table S5: Binge drinking and the interactions between period (pre-COVID-19 and during COVID-19) and age group, gender, ethnicity and education level *14*
Table S6: Alcohol frequency (drinking 4+ days per week) and the interactions between period (pre-COVID-19 and during COVID-19) and age group, gender, ethnicity and education level *16*
Table S7: Heavy drinking (5+ drinks on a typical day when drinking) and the interactions between period (pre-COVID-19 and during COVID-19) and age group, gender, ethnicity and education level *18*
Table S8: Current smoking and the interactions between period (pre-COVID-19 and during COVID-19) and age group, gender, ethnicity and education level *20*
Table S9: Regular e-cigarette use and the interactions between period (pre-COVID-19 and during COVID-19) and age group, gender, ethnicity and education level *22*

Note in the tables below:

CI: confidence interval; Ref=reference category; RR=Risk ratio

* $p < 0.05$, ** $p < 0.01$, *** $p < 0.001$

Methodological details about the creation of weights

Figure S1 shows the derivation of the analytical samples. Understanding Society began in 2009 as a stratified clustered probability sample in England, Wales and Scotland and simple probability sample of Northern Ireland. With two ethnic minority boost samples added at waves 1 and 6. Participants are followed up annually. This study begins at wave 7 (2015-2017). Within the group interviewed at each wave we excluded proxy respondents, those aged <18 years, and those who did not have a valid sampling weight. We also excluded those who had missing data on a key stratification variable (age, gender, ethnicity or education), with missing data on education being the main reason for exclusion at this stage. We further limited the sample at each wave to those with complete outcome data, but since those excluded for missing outcome data differed in terms of age, gender, ethnicity and education from those included we calculated some additional inverse probability weights (based on age, gender, ethnicity and education) within each wave to account for this. For the longitudinal models, we started with those who had full data at wave 9 (2017-19), and limited to those who participated and had valid weights and stratification data in the CA wave during April 2020 (since the CA wave sampling weights were designed to weight respondents to the CA wave to resemble the population at wave 9 during 2017-19). We then limited the longitudinal sample to those with full outcome data at all waves where outcomes were measured and calculated additional inverse probability weights to adjust for this based on wave 9 (2017-19), characteristics. Sample characteristics at wave 9 (2017-19), and how these were affected by each stage of exclusion and weighting in construction of the longitudinal sample are shown in Table S1.

Figure S1: STROBE flowcharts for cross-sectional and longitudinal analyses

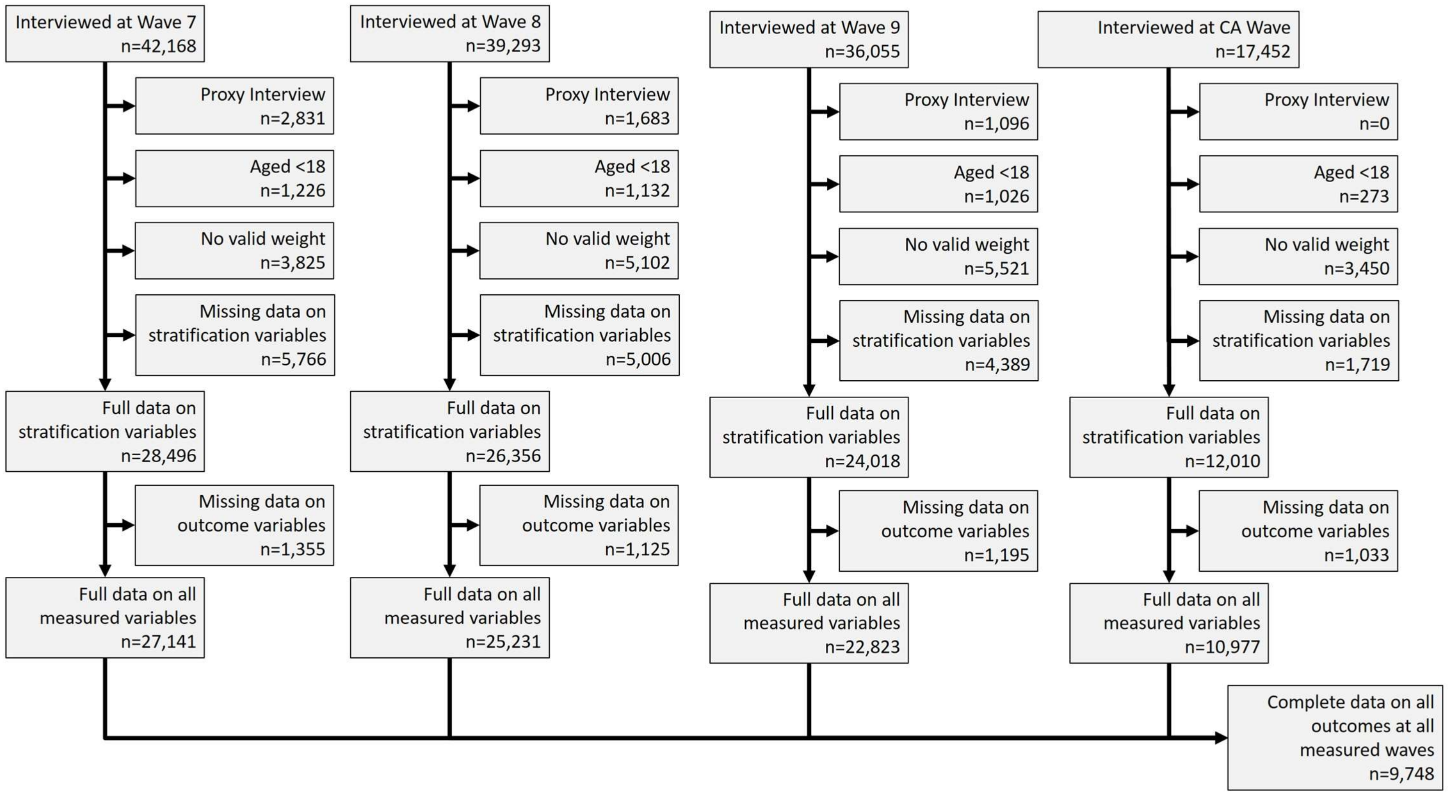

Box S1: Lockdown measures introduced in four nations of UK, March-April 2020

Information sourced from The Health Foundation's COVID-19 policy tracker for England[1], BBC News website[2], and archived Government webpages[3] accessed via the Internet Archive[4]

Date	Lockdown measure introduced	Where applicable
**12th March 2020**	All with symptoms of possible COVID-19 required to isolate for 7 days	Whole UK
**16th March 2020**	All living with someone with symptoms of possible COVID-19 required to isolate for 14 days<br><br>All others advised against unnecessary social contact and travel<br><br>Mass gatherings banned	Whole UK<br><br>Whole UK<br><br>Whole UK
**17th March 2020**	Foreign and Commonwealth Office advises against all non-essential world-wide travel	Whole UK
**20th March 2020**	Entertainment, hospitality and indoor leisure premises ordered to close<br><br>Schools, colleges and nurseries close for all except children of key workers or children identified as vulnerable by social services<br><br>**Key workers** are defined as those working in sectors considered critical to the COVID-19 response, which includes: Health and Social Care; Education and Childcare;	Whole UK<br><br>Whole UK

[1] https://www.health.org.uk/news-and-comment/charts-and-infographics/covid-19-policy-tracker
[2] www.bbc.co.uk/news
[3] https://www.gov.uk/; https://www.gov.scot/; https://gov.wales/; www.nidirect.gov.uk/
[4] https://archive.org/web/

	Key Public Services, such as Justice and Broadcasting; Local and National Government; Food and other necessary goods; Public Safety and National Security; Transport; and Utilities, Communication and Financial Services)	
**22nd March 2020**	Those identified as being extremely clinically vulnerable (i.e. those at highest risk of severe illness if they contract COVID-19) advised to begin 'shielding' – they should not leave their house for any reason, necessary food/ supplies will be provided by Local Authorities where needed<br><br>**Shielded individuals** include: solid organ transplant recipients; people with specific cancers; people with severe respiratory conditions; people who are immunosuppressed secondary to disease, treatment or medications; pregnant women with significant heart disease	Whole UK
**23rd March 2020**	Whole population not permitted to leave home except for very limited purposes (to buy food; to exercise once per day; for any medical need; to care for a vulnerable person; to travel to/from essential work)<br><br>All gatherings of more than two people in public banned<br><br>Lockdown measures to be reviewed every three weeks	Whole UK<br><br>Whole UK<br><br>Whole UK
**27th March 2020**	Public advised to only use open spaces near own house for exercise, and to stay at least 2 metres apart from other households while outdoors	Whole UK
**April 2020**	No significant change to lockdown measures	Whole UK

Table S1: Comparison of Wave 9, COVID-19 (CA) Wave and Longitudinal Sample

	**Wave 9 Analytical Sample[a] %**	**Complete Case Sample[a] %**	**Complete Case Sample, Weighted[b] %**
	n=22,823	n=9,748	n=9,748
**Age**			
**18-24**	11.6	6.4	11.7
**25-44**	29.5	29.1	30.2
**45-64**	35.3	43.3	35.5
**65+**	23.6	21.1	22.6
**Gender**			
**Men**	47.8	44.3	47.8
**Women**	52.2	55.7	52.2
**Ethnicity**			
**White**	92.1	94.9	91.5
**Asian**	4.3	2.7	4.7
**Black**	1.9	0.9	1.9
**Mixed**	1.2	1.1	1.3
**Other**	0.6	0.4	0.5
**Education**			
**Degree**	37.4	47.1	37.4
**A-Level**	11.9	11.6	12.0
**GCSE**	28.2	25.8	28.3
**None**	22.4	15.5	22.3
**Wave 9 Binge Drinking**			
**No**	89.2	90.1	89.2
**Yes**	10.8	9.9	10.8
**Wave 9 Typical Number of Drinks**			

**0-4 drinks**	86.4	88.7	87.1
**5+ drinks**	13.6	11.3	12.7
**Wave 9 Alcohol Frequency**			
**0-3 days per week**	86.3	83.8	85.2
**4+ days per week**	13.7	16.2	14.8
**Wave 9 Smoking**			
**Non-Smoker**	85.0	90.1	84.4
**<10/day**	4.9	3.0	5.0
**10-19/day**	6.6	4.6	6.9
**20+/day**	3.5	2.4	3.6
**Wave 9 Weekly E-Cigarette Use**			
**No**	95.1	95.7	94.8
**Yes**	4.9	4.3	5.2
**GHQ Case**			
**No**	80.6	82.0	80.2
**Yes**	19.4	18.0	19.8
**Loneliness**			
**No (hardly ever, never, sometimes)**	91.4	92.5	90.9
**Yes (often)**	8.6	7.5	9.1

[a]Weighted for sample representativeness and missing outcome data at wave 9.
[b]Additionally weighted for complete data at all waves.

Table S2: Mental health and health behaviours before and during the COVID-19 lockdown

	Wave 7 (2015-2017)	Wave 8 (2016-18)	Wave 9 (2017-19)	COVID-19 (CA) Wave (April 2020)
	Percentage [95% CI]	Percentage [95% CI]	Percentage [95% CI]	Percentage [95% CI]
**Psychological distress (GHQ-12, 4+ cut-off)**				
No	82.4 [81.8,83.0]	81.1 [80.5,81.8]	80.6 [79.9,81.3]	69.4 [67.8,70.9]
Yes	17.6 [17.0,18.2]	18.9 [18.2,19.5]	19.4 [18.7,20.1]	30.6 [29.1,32.3]
**Psychological distress (GHQ-12, 3+ cut-off)**				
No	78.0 [77.4,78.7]	77.0 [76.3,77.7]	76.3 [75.5,77.0]	62.0 [60.3,63.7]
Yes	22.0 [21.3,22.6]	23.0 [22.3,23.7]	23.7 [23.0,24.5]	38.0 [36.3,39.7]
**Psychological distress (GHQ-12 4+ excluding activities item)**				
No	83.6 [83.0,84.2]	82.6 [81.9,83.2]	81.8 [81.1,82.5]	73.2 [71.7,74.7]
Yes	16.4 [15.8,17.0]	17.4 [16.8,18.1]	18.2 [17.6,18.9]	26.8 [25.4,28.3]
**Loneliness**				
Never or hardly ever	-	-	62.4 [61.5,63.2]	61.3 [59.7,62.9]
Sometimes	-	-	29.0 [28.3,29.8]	29.9 [28.5,31.4]
Often	-	-	8.6 [8.1,9.1]	8.8 [7.8,9.9]
**Binge drinking (weekly or more)**				
No	88.5 [88.0,89.0]	-	89.2 [88.7,89.7]	83.8 [82.6,85.0]
Yes	11.5 [11.1,12.0]	-	10.8 [10.3,11.3]	16.2 [15.0,17.4]
**Number of drinks on typical drinking day**				
0-4	86.7	-	86.4	94.5

	[86.1,87.2]		[85.7,87.0]	[93.6,95.2]
5+	13.3<br>[12.8,13.9]	-	13.6<br>[13.0,14.3]	5.6<br>[4.8,6.4]
**Alcohol frequency**				
0-3 times per week	87.2<br>[86.6,87.7]	-	86.3<br>[85.7,86.9]	78.0<br>[76.6,79.4]
4+ times per week	12.8<br>[12.3,13.4]	-	13.7<br>[13.1,14.3]	22.0<br>[20.6,23.4]
**Cigarette smoking**				
Non-smoker	82.8<br>[82.2,83.5]	83.7<br>[83.1,84.4]	84.9<br>[84.2,85.6]	87.9<br>[86.5,89.2]
Current Smoker	17.2<br>[16.5,17.8]	16.3<br>[15.6,16.9]	15.1<br>[14.4,15.8]	12.1<br>[10.8,13.6]
**Number of cigarettes per day**				
Non-Smoker	82.9<br>[82.3,83.6]	83.8<br>[83.1,84.4]	85.0<br>[84.3,85.7]	87.9<br>[86.5,89.3]
<10	5.4<br>[5.1,5.8]	5.1<br>[4.8,5.4]	4.9<br>[4.5,5.3]	2.9<br>[2.4,3.4]
10-19	7.5<br>[7.1,8.0]	7.3<br>[6.9,7.8]	6.6<br>[6.1,7.1]	5.9<br>[5.0,7.0]
20+	4.1<br>[3.8,4.5]	3.9<br>[3.5,4.2]	3.5<br>[3.2,3.9]	3.3<br>[2.5,4.3]
**Weekly e-cigarette use**				
No	-	95.7<br>[95.3,96.1]	95.1<br>[94.7 to 95.5]	95.3<br>[94.3,96.1]
Yes	-	4.3<br>[4.0,4.7]	4.9<br>[4.5,5.3]	4.7<br>[3.9,5.7]

Table S3: Psychological distress (GHQ-12, 4+ cut-off) and the interactions between period (pre-COVID-19 and during COVID-19) and age group, gender, ethnicity and education level

	**Model 1**	**Model 2**	**Model 3**	**Model 4**
	**RR<br>[95% CI]**	**RR<br>[95% CI]**	**RR<br>[95% CI]**	**RR<br>[95% CI]**
**GHQ**				
**Year**	1.06***<br>[1.03,1.09]	1.06***<br>[1.03,1.09]	1.06***<br>[1.03,1.09]	1.05***<br>[1.03,1.09]
**Age group:**<br>18-24	2.32***<br>[1.96,2.75]	2.33***<br>[2.03,2.67]	2.33***<br>[2.03,2.67]	-
25-44	2.01***<br>[1.78,2.26]	2.01***<br>[1.83,2.22]	2.01***<br>[1.83,2.22]	1.98***<br>[1.79,2.18]
45-64	1.81***<br>[1.61,2.03]	1.71***<br>[1.55,1.88]	1.71***<br>[1.55,1.88]	1.70***<br>[1.54,1.86]
65+ (ref)				
**Gender:** Male (ref)				
Female	1.58***<br>[1.48,1.69]	1.52***<br>[1.40,1.64]	1.58***<br>[1.48,1.69]	1.58***<br>[1.48,1.69]
**Ethnicity:** White (ref)				
Non-white	1.14*<br>[1.02,1.27]	1.14*<br>[1.02,1.27]	1.16*<br>[1.01,1.33]	1.18**<br>[1.05,1.32]
**Period:** Pre-COVID-19 (ref)				
During COVID-19	1.35***<br>[1.16,1.57]	1.18*<br>[1.04,1.34]	1.29***<br>[1.16,1.43]	1.43***<br>[1.27,1.61]
**Age group interaction with period (Pre-COVID-19 is ref):**<br>a18to24 # During COVID-19	1.00<br>[0.81,1.24]			
a25to44 # During COVID-19	1.01<br>[0.88,1.17]			
a45to64 # During COVID-19	0.84*<br>[0.74,0.97]			
a65over # During COVID-19 (ref)				
**Gender interaction with period (Pre-COVID-19 /male**				

**is ref):** Male # During COVID-19				
Female # During COVID-19		1.14* [1.03,1.26]		
**Ethnicity interaction with period (Pre-COVID-19 / white is ref):** White # During COVID-19				
Non-white # During COVID-19			0.94 [0.78,1.14]	
**Education level:** Degree (ref)				
ALevel				1.08 [0.95,1.23]
GCSE				1.01 [0.92,1.11]
None				1.05 [0.94,1.17]
**Education level interaction with period (Pre-COVID-19 / Degree is ref):** Degree # During COVID-19				
ALevel # During COVID-19				0.84* [0.72,0.99]
GCSE # During COVID-19				0.86** [0.77,0.96]
None # During COVID-19				0.76*** [0.67,0.87]
**Observations**	38992	38992	38992	36984

Model 1: Year, age group, gender, ethnicity, period and period x age group interaction

Model 2: Year, age group, gender, ethnicity, period and period x gender interaction

Model 3: Year, age group, gender, ethnicity, period and period x ethnicity interaction

Model 4: Year, age group, gender, ethnicity, period, education level and period x education level interaction (sample limited to individuals aged 25+ years)

Table S4: Loneliness and the interactions between period (pre-COVID-19 and during COVID-19) and age group, gender, ethnicity and education level

	**Model 1**	**Model 2**	**Model 3**	**Model 4**
**Loneliness**	**RR**<br>**[95% CI]_**	**RR**<br>**[95% CI]_**	**RR**<br>**[95% CI]**	**RR**<br>**[95% CI]**
**Year**	1.00<br>[0.82,1.21]	1.00<br>[0.83,1.20]	0.99<br>[0.82,1.20]	0.91<br>[0.77,1.07]
**Age group:**<br>18-24	4.89^{***}<br>[3.33,7.20]	4.76^{***}<br>[3.55,6.39]	4.76^{***}<br>[3.55,6.39]	
25-44	3.01^{***}<br>[2.23,4.06]	2.73^{***}<br>[2.15,3.46]	2.73^{***}<br>[2.15,3.45]	2.79^{***}<br>[2.19,3.56]
45-64	2.12^{***}<br>[1.58,2.84]	2.02^{***}<br>[1.61,2.54]	2.02^{***}<br>[1.61,2.54]	2.02^{***}<br>[1.60,2.54]
65+ (ref)				
**Gender:** Male (ref)				
Female	1.64^{***}<br>[1.41,1.91]	1.42^{***}<br>[1.17,1.71]	1.64^{***}<br>[1.41,1.91]	1.72^{***}<br>[1.46,2.02]
**Ethnicity:** White (ref)				
Non-white	1.12<br>[0.88,1.44]	1.12<br>[0.88,1.44]	1.26<br>[0.93,1.70]	1.37^{*}<br>[1.06,1.77]
**Period:** Pre-COVID-19 (ref)				
During COVID-19	1.01<br>[0.58,1.76]	0.74<br>[0.44,1.25]	0.94<br>[0.56,1.56]	1.26<br>[0.82,1.92]
**Age group interaction with period (Pre-COVID-19 is ref):** a18to24 # During COVID-19	0.95<br>[0.59,1.53]			
a25to44 # During COVID-19	0.81<br>[0.57,1.16]			
a45to64 # During COVID-19	0.90<br>[0.64,1.28]			
a65over # During COVID-19 (ref)				
**Gender interaction with period (Pre-COVID-19 /male is ref):** Male # During				

COVID-19				
Female # During COVID-19		1.37* [1.07,1.77]		
**Ethnicity interaction with period (Pre-COVID-19 / white is ref):** White # During COVID-19				
Non-white # During COVID-19			0.78 [0.52,1.15]	
**Education level:** Degree (ref)				
ALevel				1.34 [0.98,1.83]
GCSE				1.52*** [1.21,1.90]
None				1.45** [1.12,1.88]
**Education level interaction with period (Pre-COVID-19 / Degree is ref):** Degree # During COVID-19				
ALevel # During COVID-19				0.91 [0.62,1.36]
GCSE # During COVID-19				0.78 [0.59,1.03]
None # During COVID-19				0.87 [0.66,1.15]
**Observations**	19496	19496	19496	18492

Model 1: Year, age group, gender, ethnicity, period and period x age group interaction

Model 2: Year, age group, gender, ethnicity, period and period x gender interaction

Model 3: Year, age group, gender, ethnicity, period and period x ethnicity interaction

Model 4: Year, age group, gender, ethnicity, period, education level and period x education level interaction (sample limited to individuals aged 25+ years)

Table S5: Binge drinking and the interactions between period (pre-COVID-19 and during COVID-19) and age group, gender, ethnicity and education level

	Model 1	Model 2	Model 3	Model 4
**Binge drinking**	RR [95% CI]	RR [95% CI]	RR [95% CI]	RR [95% CI]
**Year**	1.02 [0.98,1.06]	1.02 [0.98,1.06]	1.02 [0.98,1.06]	0.99 [0.95,1.02]
**Age group:** 18-24	1.55** [1.18,2.04]	1.59*** [1.25,2.01]	1.59*** [1.25,2.01]	
25-44	1.39*** [1.17,1.66]	1.73*** [1.50,2.01]	1.73*** [1.50,2.01]	1.73*** [1.49,2.00]
45-64	1.87*** [1.60,2.18]	1.98*** [1.74,2.26]	1.98*** [1.74,2.26]	1.95*** [1.71,2.22]
65+ (ref)				
**Gender:** Male (ref)				
Female	0.53*** [0.49,0.59]	0.45*** [0.40,0.50]	0.53*** [0.49,0.59]	0.52*** [0.47,0.57]
**Ethnicity:** White (ref)				
Non-white	0.27*** [0.20,0.36]	0.27*** [0.20,0.36]	0.23*** [0.16,0.34]	0.30*** [0.22,0.41]
**Period:** Pre-COVID-19 (ref)				
During COVID-19	1.18 [0.97,1.45]	1.27** [1.08,1.48]	1.47*** [1.25,1.71]	1.95*** [1.67,2.27]
**Age group interaction with period (Pre-COVID-19 is ref):** a18to24 # During COVID-19	1.06 [0.77,1.46]			
a25to44 # During COVID-19	1.64*** [1.35,1.98]			
a45to64 # During COVID-19	1.16 [0.98,1.37]			
a65over # During COVID-19 (ref)				
**Gender interaction with period (Pre-COVID-19 /male is ref):** Male # During COVID-19				
Female # During COVID-19		1.49***		

		[1.31,1.69]		
**Ethnicity interaction with period (Pre-COVID-19 / white is ref):** White # During COVID-19				
Non-white # During COVID-19			1.37 [0.88,2.15]	
**Education level:** Degree (ref)				
ALevel				1.18 [0.96,1.46]
GCSE				1.29*** [1.12,1.49]
None				1.20* [1.02,1.42]
**Education level interaction with period (Pre-COVID-19 / Degree is ref):** Degree # During COVID-19				
ALevel # During COVID-19				0.85 [0.69,1.03]
GCSE # During COVID-19				0.85* [0.74,0.98]
None # During COVID-19				0.69*** [0.59,0.82]
**Observations**	29244	29244	29244	27738

Model 1: Year, age group, gender, ethnicity, period and period x age group interaction

Model 2: Year, age group, gender, ethnicity, period and period x gender interaction

Model 3: Year, age group, gender, ethnicity, period and period x ethnicity interaction

Model 4: Year, age group, gender, ethnicity, period, education level and period x education level interaction (sample limited to individuals aged 25+ years)

Table S6: Alcohol frequency (drinking 4+ days per week) and the interactions between period (pre-COVID-19 and during COVID-19) and age group, gender, ethnicity and education level

	Model 1	Model 2	Model 3	Model 4
**Drinking 4+ days per week**	RR [95% CI]	RR [95% CI]	RR [95% CI]	RR [95% CI]
**Year**	1.05*** [1.03,1.08]	1.05*** [1.03,1.08]	1.05*** [1.03,1.08]	1.04** [1.02,1.06]
**Age group:** 18-24	0.10*** [0.06,0.16]	0.17*** [0.13,0.23]	0.17*** [0.13,0.23]	
25-44	0.35*** [0.31,0.41]	0.48*** [0.43,0.54]	0.48*** [0.43,0.54]	0.44*** [0.40,0.50]
45-64	0.79*** [0.71,0.88]	0.87** [0.79,0.95]	0.87** [0.79,0.95]	0.84*** [0.77,0.92]
65+ (ref)				
**Gender:** Male (ref)				
Female	0.62*** [0.57,0.67]	0.54*** [0.49,0.59]	0.62*** [0.57,0.67]	0.62*** [0.57,0.67]
**Ethnicity:** White (ref)				
Non-white	0.34*** [0.26,0.43]	0.34*** [0.26,0.43]	0.34*** [0.25,0.47]	0.33*** [0.26,0.43]
**Period:** Pre-COVID-19 (ref)				
During COVID-19	1.06 [0.96,1.17]	1.23*** [1.11,1.35]	1.38*** [1.26,1.51]	1.44*** [1.31,1.58]
**Age group interaction with period (Pre-COVID-19 is ref):** a18to24 # During COVID-19	2.90*** [1.63,5.15]			
a25to44 # During COVID-19	1.91*** [1.66,2.20]			
a45to64 # During COVID-19	1.25*** [1.15,1.35]			
a65over # During COVID-19 (ref)				
**Gender interaction with period (Pre-COVID-19 /male is ref):** Male # During COVID-19				
Female # During COVID-19		1.34*** [1.23,1.47]		

**Ethnicity interaction with period (Pre-COVID-19 / white is ref):** White # During COVID-19				
Non-white # During COVID-19			0.98 [0.67,1.42]	
**Education level:** Degree (ref)				
ALevel				0.81* [0.69,0.96]
GCSE				0.71*** [0.63,0.79]
None				0.60*** [0.52,0.68]
**Education level interaction with period (Pre-COVID-19 / Degree is ref):** Degree # During COVID-19				
ALevel # During COVID-19				0.96 [0.82,1.12]
GCSE # During COVID-19				0.95 [0.86,1.05]
None # During COVID-19				0.96 [0.86,1.08]
**Observations**	29244	29244	29244	27738

Model 1: Year, age group, gender, ethnicity, period and period x age group interaction

Model 2: Year, age group, gender, ethnicity, period and period x gender interaction

Model 3: Year, age group, gender, ethnicity, period and period x ethnicity interaction

Model 4: Year, age group, gender, ethnicity, period, education level and period x education level interaction (sample limited to individuals aged 25+ years)

Table S7: Heavy drinking (5+ drinks on a typical day when drinking) and the interactions between period (pre-COVID-19 and during COVID-19) and age group, gender, ethnicity and education level

	**Model 1**	**Model 2**	**Model 3**	**Model 4**
**5+ drinks on typical day**	RR [95% CI]	RR [95% CI]	RR [95% CI]	RR [95% CI]
**Year**	1.02 [0.98,1.06]	1.02 [0.98,1.06]	1.02 [0.98,1.06]	0.98 [0.94,1.01]
**Age group:** 18-24	18.04*** [13.65,23.83]	15.29*** [11.73,19.93]	15.29*** [11.73,19.92]	
25-44	8.26*** [6.48,10.54]	7.62*** [6.06,9.59]	7.62*** [6.06,9.58]	8.03*** [6.36,10.13]
45-64	4.62*** [3.62,5.91]	4.46*** [3.55,5.62]	4.46*** [3.55,5.62]	4.45*** [3.53,5.62]
65+ (ref)				
**Gender:** Male (ref)				
Female	0.52*** [0.46,0.59]	0.56*** [0.49,0.63]	0.52*** [0.46,0.59]	0.49*** [0.43,0.56]
**Ethnicity:** White (ref)				
Non-white	0.30*** [0.22,0.40]	0.30*** [0.22,0.40]	0.33*** [0.24,0.45]	0.32*** [0.23,0.46]
**Period:** Pre-COVID-19 (ref)				
During COVID-19	0.60** [0.42,0.86]	0.46*** [0.38,0.55]	0.41*** [0.35,0.49]	0.58*** [0.48,0.72]
**Age group interaction with period (Pre-COVID-19 is ref):** a18to24 # During COVID-19	0.37*** [0.22,0.63]			
a25to44 # During COVID-19	0.68* [0.47,0.99]			
a45to64 # During COVID-19	0.86 [0.60,1.23]			
a65over # During COVID-19 (ref)				
**Gender interaction with period (Pre-COVID-19 /male is ref):** Male # During COVID-19				
Female # During COVID-19		0.70**		

		[0.56,0.88]		
**Ethnicity interaction with period (Pre-COVID-19 / white is ref):** White # During COVID-19				
Non-white # During COVID-19			0.53<br>[0.23,1.20]	
**Education level:** Degree (ref)				
ALevel				1.43**<br>[1.14,1.78]
GCSE				1.67***<br>[1.43,1.96]
None				1.33**<br>[1.09,1.62]
**Education level interaction with period (Pre-COVID-19 / Degree is ref):** Degree # During COVID-19				
ALevel # During COVID-19				0.92<br>[0.66,1.29]
GCSE # During COVID-19				0.85<br>[0.66,1.09]
None # During COVID-19				0.90<br>[0.68,1.20]
**Observations**	29244	29244	29244	27738

Model 1: Year, age group, gender, ethnicity, period and period x age group interaction

Model 2: Year, age group, gender, ethnicity, period and period x gender interaction

Model 3: Year, age group, gender, ethnicity, period and period x ethnicity interaction

Model 4: Year, age group, gender, ethnicity, period, education level and period x education level interaction (sample limited to individuals aged 25+ years)

Table S8: Current smoking and the interactions between period (pre-COVID-19 and during COVID-19) and age group, gender, ethnicity and education level

	**Model 1**	**Model 2**	**Model 3**	**Model 4**
**Current smoking**	RR [95% CI]	RR [95% CI]	RR [95% CI]	RR [95% CI]
**Year**	0.99 [0.97,1.01]	0.99 [0.97,1.01]	0.99 [0.97,1.01]	0.98 [0.96,1.00]
**Age group:** 18-24	2.72*** [1.54,4.81]	2.85*** [1.62,5.02]	2.85*** [1.62,5.02]	
25-44	6.46*** [4.34,9.60]	6.56*** [4.42,9.75]	6.56*** [4.42,9.75]	7.26*** [5.14,10.28]
45-64	5.14*** [3.46,7.65]	5.29*** [3.56,7.87]	5.29*** [3.56,7.87]	4.96*** [3.52,6.98]
65+ (ref)				
**Gender:** Male (ref)				
Female	1.00 [0.82,1.22]	0.99 [0.81,1.21]	1.00 [0.82,1.22]	1.01 [0.83,1.22]
**Ethnicity:** White (ref)				
Non-white	1.19 [0.88,1.61]	1.19 [0.88,1.61]	1.20 [0.88,1.63]	1.54** [1.14,2.08]
**Period:** Pre-COVID-19 (ref)				
During COVID-19	0.80** [0.69,0.93]	0.88* [0.78,0.98]	0.90* [0.82,0.98]	0.90 [0.80,1.02]
**Age group interaction with period (Pre-COVID-19 is ref):** a18to24 # During COVID-19	1.23 [0.95,1.60]			
a25to44 # During COVID-19	1.08 [0.91,1.28]			
a45to64 # During COVID-19	1.14 [0.99,1.32]			
a65over # During COVID-19 (ref)				
**Gender interaction with period (Pre-COVID-19 /male is ref):** Male # During COVID-19				
Female # During COVID-19		1.03 [0.93,1.16]		

**Ethnicity interaction with period (Pre-COVID-19 / white is ref):** White # During COVID-19				
Non-white # During COVID-19			0.95<br>[0.74,1.22]	
**Education level:** Degree (ref)				
ALevel				2.64***<br>[1.89,3.69]
GCSE				3.84***<br>[3.03,4.87]
None				4.43***<br>[3.40,5.77]
**Education level interaction with period (Pre-COVID-19 / Degree is ref):** Degree # During COVID-19				
ALevel # During COVID-19				0.93<br>[0.76,1.14]
GCSE # During COVID-19				1.10<br>[0.98,1.23]
None # During COVID-19				0.92<br>[0.78,1.08]
**Observations**	38992	38992	38992	36984

Model 1: Year, age group, gender, ethnicity, period and period x age group interaction

Model 2: Year, age group, gender, ethnicity, period and period x gender interaction

Model 3: Year, age group, gender, ethnicity, period and period x ethnicity interaction

Model 4: Year, age group, gender, ethnicity, period, education level and period x education level interaction (sample limited to individuals aged 25+ years)

Table S9: Regular e-cigarette use and the interactions between period (pre-COVID-19 and during COVID-19) and age group, gender, ethnicity and education level

	**Model 1**	**Model 2**	**Model 3**	**Model 4**
**Regular e-cigarettes**	RR [95% CI]	RR [95% CI]	RR [95% CI]	RR [95% CI]
**Year**	1.11* [1.00,1.22]	1.10 [1.00,1.22]	1.10* [1.00,1.21]	1.08 [0.98,1.19]
**Age group:** 18-24	2.04 [0.89,4.64]	2.04 [0.95,4.39]	2.04 [0.95,4.39]	
25-44	6.40*** [4.19,9.75]	6.23*** [4.14,9.37]	6.23*** [4.14,9.37]	6.94*** [4.58,10.51]
45-64	3.94*** [2.67,5.82]	3.94*** [2.70,5.74]	3.94*** [2.70,5.74]	3.99*** [2.71,5.88]
65+ (ref)				
**Gender:** Male (ref)				
Female	0.76* [0.58,0.99]	0.71* [0.54,0.95]	0.76* [0.58,0.99]	0.74* [0.56,0.96]
**Ethnicity:** White (ref)				
Non-white	0.52* [0.32,0.86]	0.52* [0.32,0.86]	0.57 [0.32,1.02]	0.52* [0.31,0.88]
**Period:** Pre-COVID-19 (ref)				
During COVID-19	0.68 [0.46,1.01]	0.61** [0.43,0.86]	0.68** [0.51,0.91]	0.84 [0.60,1.17]
**Age group interaction with period (Pre-COVID-19 is ref):** a18to24 # During COVID-19	1.01 [0.40,2.58]			
a25to44 # During COVID-19	0.92 [0.64,1.31]			
a45to64 # During COVID-19	1.00 [0.71,1.39]			
a65over # During COVID-19 (ref)				
**Gender interaction with period (Pre-COVID-19 /male is ref):** Male # During COVID-19				
Female # During COVID-19		1.20 [0.91,1.58]		
**Ethnicity interaction with period (Pre-COVID-19 / white is**				

**ref):** White # During COVID-19				
Non-white # During COVID-19			0.73 [0.22,2.43]	
**Education level:** Degree (ref)				
ALevel				1.95** [1.21,3.14]
GCSE				2.76*** [1.99,3.84]
None				2.40*** [1.62,3.57]
**Education level interaction with period (Pre-COVID-19 / Degree is ref):** Degree # During COVID-19				
ALevel # During COVID-19				0.79 [0.53,1.16]
GCSE # During COVID-19				0.82 [0.65,1.05]
None # During COVID-19				0.75 [0.55,1.01]
**Observations**	29244	29244	29244	27738

Model 1: Year, age group, gender, ethnicity, period and period x age group interaction

Model 2: Year, age group, gender, ethnicity, period and period x gender interaction

Model 3: Year, age group, gender, ethnicity, period and period x ethnicity interaction

Model 4: Year, age group, gender, ethnicity, period, education level and period x education level interaction (sample limited to individuals aged 25+ years)
