# Supplementary material for: Mental health and health behaviours before and during the initial phase of the COVID-19 lockdown: longitudinal analyses of the UK Household Longitudinal Study
Source: J Epidemiol Community Health. 2020 Sep 24;75(3):224–31. doi: 10.1136/jech-2020-215060 (PMC7892383; doi:10.1136/jech-2020-215060)
Supplement: Supplementary data [file jech-2020-215060supp002.pdf]

APPENDIX 2		2015-17			2016-18			2017-19			April 2020		
**GHQ-12 Items**		**Wave 7**			**Wave 8**			**Wave 9**			**CA Wave**		
GHQ Problem concentrating		**Percentage**	**Lower 95% CI**	**Upper 95% CI**	**Percentage**	**Lower 95% CI**	**Upper 95% CI**	**Percentage**	**Lower 95% CI**	**Upper 95% CI**	**Percentage**	**Lower 95% CI**	**Upper 95% CI**
	No	**83.9**	83.3	84.4	**83.5**	82.9	84.1	**83.2**	82.6	83.8	**69.9**	68.3	71.5
	Yes	**16.2**	15.6	16.7	**16.5**	15.9	17.1	**16.8**	16.2	17.5	**30.1**	28.5	31.7
GHQ Loss of sleep													
	No	**85.6**	85.1	86.1	**83.5**	82.9	84.1	**83.6**	83.0	84.3	**74.8**	73.3	76.2
	Yes	**14.4**	13.9	14.9	**16.5**	15.9	17.1	**16.4**	15.7	17.0	**25.2**	23.8	26.7
GHQ Not feeling useful													
	No	**86.6**	86.1	87.1	**85.9**	85.4	86.5	**85.0**	84.3	85.6	**72.3**	70.8	73.8
	Yes	**13.4**	12.9	13.9	**14.1**	13.5	14.6	**15.0**	14.5	15.7	**27.7**	26.2	29.2
GHQ Less capable of making decisions													
	No	**91.2**	90.7	91.6	**90.8**	90.4	91.3	**90.1**	89.6	90.6	**86.0**	84.8	87.2
	Yes	**8.8**	8.4	9.3	**9.2**	8.7	9.6	**9.9**	9.4	10.4	**14.0**	12.8	15.2
GHQ Constantly under strain													
	No	**78.1**	77.5	78.7	**78.6**	77.9	79.2	**77.2**	76.5	77.9	**71.5**	70.0	72.9
	Yes	**21.9**	21.3	22.5	**21.4**	20.8	22.1	**22.8**	22.1	23.5	**28.5**	27.1	30.0
GHQ Problem overcoming difficulties													
	No	**86.8**	86.3	87.3	**86.3**	85.7	86.8	**85.5**	84.9	86.1	**83.9**	82.8	85.1
	Yes	**13.2**	12.7	13.7	**13.7**	13.2	14.3	**14.5**	13.9	15.1	**16.1**	14.9	17.2
GHQ Less able to enjoy day-to-day activities													
	No	**83.8**	83.2	84.3	**83.2**	82.6	83.7	**82.9**	82.3	83.6	**54.6**	52.8	56.4
	Yes	**16.2**	15.7	16.8	**16.8**	16.3	17.4	**17.1**	16.5	17.7	**45.4**	43.6	47.2
GHQ Less able to face problems													
	No	**90.2**	89.8	90.6	**89.5**	89.1	90.0	**89.4**	88.8	89.9	**85.5**	84.2	86.6
	Yes	**9.8**	9.4	10.3	**10.5**	10.0	11.0	**10.6**	10.1	11.2	**14.5**	13.4	15.8
GHQ Unhappy or depressed													
	No	**81.2**	80.6	81.8	**80.6**	79.9	81.2	**80.0**	79.3	80.7	**70.4**	68.9	71.9
	Yes	**18.8**	18.2	19.4	**19.5**	18.8	20.1	**20.0**	19.3	20.7	**29.6**	28.1	31.2
GHQ Losing confidence													
	No	**84.8**	84.3	85.4	**84.0**	83.4	84.6	**83.8**	83.2	84.4	**80.7**	79.3	82.0
	Yes	**15.2**	14.7	15.7	**16.0**	15.4	16.6	**16.2**	15.6	16.8	**19.3**	18.0	20.7
GHQ Feeling worthless													
	No	**91.6**	91.2	92.0	**91.0**	90.5	91.5	**90.8**	90.3	91.3	**88.5**	87.3	89.6
	Yes	**8.4**	8.0	8.8	**9.0**	8.5	9.5	**9.2**	8.7	9.7	**11.5**	10.4	12.7
GHQ Feeling less happy													
	No	**86.4**	85.9	87.0	**86.0**	85.5	86.6	**85.2**	84.6	85.8	**75.9**	74.4	77.4
	Yes	**13.6**	13.1	14.1	**14.0**	13.4	14.5	**14.8**	14.2	15.4	**24.1**	22.6	25.6

GHQ-12 case (4+ cut-off)	Wave 7			Wave 8			Wave 9			CA Wave		
	Percentage	Lower 95% CI	Upper 95% CI	Percentage	Lower 95% CI	Upper 95% CI	Percentage	Lower 95% CI	Upper 95% CI	Percentage	Lower 95% CI	Upper 95% CI
**Age group**												
18-24	**20.5**	18.7	22.4	**20.3**	18.4	22.5	**24.0**	21.9	26.3	**44.0**	38.6	49.6
25-44	**19.4**	18.3	20.5	**20.3**	19.1	21.5	**22.0**	20.7	23.4	**37.8**	34.8	40.9
45-64	**18.2**	17.3	19.1	**19.8**	18.8	20.8	**20.1**	19.1	21.2	**28.1**	26.0	30.3
65+	**12.8**	11.8	13.9	**14.9**	14.0	16.0	**12.7**	11.7	13.7	**19.2**	16.5	22.2
**Gender**												
Male	**14.7**	13.9	15.5	**15.8**	14.9	16.6	**15.5**	14.6	16.5	**24.0**	21.7	26.5
Female	**20.3**	19.5	21.0	**21.7**	20.9	22.6	**23.0**	22.0	23.9	**36.8**	34.8	38.8
**Ethnicity**												
White	**17.5**	16.9	18.1	**18.8**	18.2	19.5	**19.2**	18.5	20.0	**30.2**	28.6	31.9
Asian	**17.6**	15.6	19.7	**16.8**	14.6	19.2	**18.7**	16.4	21.2	**34.9**	27.3	43.2
Black	**19.6**	16.6	22.8	**19.1**	15.7	23.0	**21.2**	16.4	26.8	**29.2**	14.7	49.8
Mixed	**21.8**	17.3	27.2	**27.2**	21.5	33.6	**30.8**	24.3	38.1	**39.7**	23.9	58.0
Other	**19.0**	13.4	26.2	**25.6**	17.2	36.3	**23.9**	15.4	35.1	**40.9**	21.9	63.2
**Education level**												
Degree	**16.3**	15.4	17.2	**17.3**	16.4	18.4	**18.0**	17.0	19.1	**30.6**	28.8	32.5
ALevel	**17.6**	15.7	19.7	**19.7**	17.5	22.1	**20.7**	18.5	23.1	**30.7**	26.7	35.0
GCSE	**17.6**	16.5	18.8	**19.0**	17.9	20.2	**19.5**	18.2	21.0	**28.6**	25.5	31.9
None	**18.0**	16.9	19.2	**20.1**	18.8	21.4	**18.5**	17.2	19.9	**25.5**	20.4	31.2

**Loneliness**		**Wave 9**			**CA Wave**		
		**Percentage**	**Lower 95% CI**	**Upper 95% CI**	**Percentage**	**Lower 95% CI**	**Upper 95% CI**
**Age group**							
	18-24	**13.3**	11.6	15.3	**20.2**	16.0	25.2
	25-44	**10.0**	9.1	11.0	**9.8**	8.1	11.7
	25-64	**7.9**	7.2	8.7	**7.3**	5.9	9.0
	65+	**5.5**	4.8	6.3	**4.6**	3.2	6.5
**Gender**							
	Male	**7.6**	7.0	8.3	**6.5**	5.3	8.0
	Female	**9.5**	8.8	10.2	**10.9**	9.5	12.5
**Ethnicity**							
	White	**8.5**	8.0	9.1	**8.7**	7.7	9.8
	Asian	**8.3**	6.8	10.2	**9.6**	6.5	14.0
	Black	**8.4**	6.0	11.7	**5.4**	2.5	11.2
	Mixed	**15.1**	10.2	21.7	**16.9**	4.8	45.1
	Other	**8.2**	2.6	23.2	**5.3**	0.6	34.1
**Education level**							
	Degree	**6.3**	5.6	7.0	**7.0**	5.7	8.6
	ALevel	**9.0**	7.5	10.8	**8.1**	5.8	11.2
	GCSE	**9.4**	8.4	10.4	**6.5**	5.3	8.1
	None	**8.9**	7.9	9.9	**9.2**	6.5	12.9

Binge drinking	Wave 7			Wave 9			CA Wave		
	Percentage	Lower 95% CI	Upper 95% CI	Percentage	Lower 95% CI	Upper 95% CI	Percentage	Lower 95% CI	Upper 95% CI
**Age group**									
18-24	**13.1**	11.6	14.7	**12.6**	11.0	14.5	**12.6**	9.2	17.1
25-44	**11.7**	10.8	12.6	**11.2**	10.2	12.2	**19.7**	17.5	22.1
45-64	**13.7**	12.9	14.6	**12.6**	11.7	13.5	**18.1**	16.5	19.8
65+	**7.2**	6.5	8.0	**6.7**	6.0	7.6	**10.1**	8.1	12.5
**Gender**									
Male	**16.0**	15.2	16.8	**14.3**	13.4	15.1	**18.8**	16.9	20.7
Female	**7.4**	6.9	7.9	**7.6**	7.1	8.2	**13.8**	12.6	15.0
**Ethnicity**									
White	**12.2**	11.7	12.8	**11.4**	10.8	12.0	**17.2**	16.0	18.5
Asian	**1.9**	1.3	2.6	**2.8**	1.6	4.8	**2.2**	1.3	3.6
Black	**3.5**	2.5	4.8	**4.2**	2.6	6.5	**5.0**	2.7	9.2
Mixed	**8.7**	5.9	12.8	**7.7**	4.9	11.8	**14.5**	7.3	26.7
Other	**3.4**	1.6	7.5	**2.8**	0.8	9.8	**16.4**	8.1	30.4
**Education level**									
Degree	**9.3**	8.6	10.1	**9.2**	8.4	10.0	**17.1**	15.5	18.8
ALevel	**13.0**	11.3	14.9	**11.0**	9.3	13.0	**18.4**	15.2	22.1
GCSE	**13.6**	12.6	14.6	**12.7**	11.7	13.9	**18.6**	16.3	21.2
None	**11.4**	10.4	12.4	**10.2**	9.2	11.3	**11.7**	9.6	14.3

Drinking 4+ times per week	Wave 7			Wave 9			CA Wave		
	Percentage	Lower 95% CI	Upper 95% CI	Percentage	Lower 95% CI	Upper 95% CI	Percentage	Lower 95% CI	Upper 95% CI
**Age group**									
18-24	**3.8**	3.0	4.9	**4.4**	3.4	5.6	**9.5**	7.0	12.7
25-44	**8.2**	7.5	9.0	**8.0**	7.1	8.9	**17.7**	15.6	20.0
45-64	**16.4**	15.5	17.4	**16.9**	15.9	17.9	**24.7**	22.7	26.9
65+	**17.9**	16.7	19.1	**20.7**	19.4	22.1	**28.8**	25.5	32.4
**Gender**									
Male	**16.8**	16.0	17.6	**17.6**	16.7	18.6	**26.0**	23.7	28.4
Female	**9.2**	8.6	9.8	**10.1**	9.5	10.7	**18.2**	16.9	19.6
**Ethnicity**									
White	**13.6**	13.1	14.2	**14.5**	13.9	15.2	**23.6**	22.1	25.2
Asian	**2.3**	1.7	3.1	**2.9**	2.1	4.0	**2.9**	1.8	4.7
Black	**3.4**	2.4	4.8	**3.0**	2.0	4.5	**8.0**	4.6	13.6
Mixed	**9.8**	6.4	14.9	**6.4**	3.9	10.2	**8.0**	4.0	15.4
Other	**3.6**	1.5	8.7	**9.4**	4.8	17.7	**19.1**	9.7	34.4
**Education level**									
Degree	**16.3**	15.4	17.3	**17.0**	16.0	18.1	**27.1**	25.2	29.1
ALevel	**14.8**	13.0	16.7	**14.2**	12.3	16.2	**21.6**	18.2	25.4
GCSE	**12.9**	11.9	13.9	**14.1**	13.0	15.2	**20.9**	18.3	23.8
None	**11.3**	10.4	12.4	**12.8**	11.7	14.1	**20.5**	16.6	25.0

5+ drinks on typical drinking day	Wave 7			Wave 9			CA Wave		
	Percentage	Lower 95% CI	Upper 95% CI	Percentage	Lower 95% CI	Upper 95% CI	Percentage	Lower 95% CI	Upper 95% CI
**Age group**									
18-24	**32.8**	30.5	35.1	**31.9**	29.5	34.5	**8.5**	5.4	13.2
25-44	**17.9**	16.8	19.1	**18.1**	16.8	19.5	**6.9**	5.4	8.9
45-64	**10.3**	9.6	11.1	**11.2**	10.3	12.1	**6.0**	5.1	7.0
65+	**2.6**	2.2	3.1	**2.7**	2.2	3.2	**1.7**	1.2	2.3
**Gender**									
Male	**17.1**	16.2	18.0	**16.8**	15.8	17.8	**7.6**	6.3	9.2
Female	**9.9**	9.3	10.5	**10.7**	10.0	11.5	**3.6**	3.1	4.3
**Ethnicity**									
White	**14.0**	13.4	14.6	**14.2**	13.5	14.9	**5.8**	5.0	6.6
Asian	**3.4**	2.2	5.3	**3.9**	2.6	5.9	**0.6**	0.3	1.3
Black	**5.7**	3.6	8.8	**6.3**	3.5	11.1	**0.8**	0.4	1.7
Mixed	**18.2**	13.5	24.1	**21.7**	15.2	29.9	**15.2**	5.4	36.0
Other	**6.0**	2.5	13.8	**9.0**	4.1	18.5	**2.7**	0.3	22.9
**Education level**									
Degree	**8.3**	7.6	9.1	**9.2**	8.4	10.1	**4.6**	3.8	5.4
ALevel	**14.8**	13.0	16.8	**14.7**	12.7	17.0	**6.4**	4.8	8.5
GCSE	**14.4**	13.4	15.5	**14.2**	13.0	15.5	**6.5**	4.9	8.7
None	**9.9**	9.0	10.8	**9.9**	8.8	11.1	**4.0**	2.8	5.5

Current cigarette smoking	Wave 7			Wave 8			Wave 9			CA Wave		
	Percentage	Lower 95% CI	Upper 95% CI	Percentage	Lower 95% CI	Upper 95% CI	Percentage	Lower 95% CI	Upper 95% CI	Percentage	Lower 95% CI	Upper 95% CI
**Age group**												
18-24	**19.4**	17.5	21.5	**18.7**	16.7	20.8	**16.2**	14.3	18.4	**9.9**	7.0	14.0
25-44	**21.6**	20.4	22.9	**20.1**	18.8	21.5	**19.0**	17.6	20.5	**15.5**	12.9	18.3
45-64	**18.0**	17.0	19.0	**17.0**	16.0	18.1	**16.1**	15.0	17.2	**14.5**	12.1	17.3
65+	**8.9**	8.1	9.8	**9.0**	8.2	10.0	**8.1**	7.2	9.0	**4.8**	3.6	6.3
**Gender**												
Male	**18.1**	17.2	18.9	**17.1**	16.2	18.0	**15.7**	14.7	16.7	**12.0**	10.1	14.2
Female	**16.3**	15.6	17.1	**15.6**	14.8	16.4	**14.5**	13.7	15.3	**12.2**	10.5	14.0
**Ethnicity**												
White	**17.6**	16.9	18.3	**16.7**	16.0	17.5	**15.4**	14.6	16.2	**12.0**	10.7	13.6
Asian	**8.2**	6.9	9.8	**7.8**	6.5	9.3	**8.4**	6.9	10.3	**5.2**	3.3	8.3
Black	**12.7**	10.2	15.7	**12.9**	10.1	16.3	**11.5**	8.4	15.6	**24.7**	9.5	50.8
Mixed	**22.7**	17.9	28.4	**18.7**	14.0	24.5	**21.8**	15.6	29.7	**16.6**	8.0	31.1
Other	**15.9**	10.0	24.3	**13.6**	7.4	23.8	**11.6**	5.6	22.7	**24.7**	13.1	41.7
**Education level**												
Degree	**8.8**	8.1	9.6	**8.2**	7.5	9.0	**7.7**	7.0	8.5	**6.0**	5.0	7.2
ALevel	**16.9**	15.0	19.0	**15.3**	13.4	17.4	**14.4**	12.5	16.7	**11.1**	8.3	14.7
GCSE	**22.7**	21.4	24.0	**21.6**	20.3	23.0	**20.2**	18.8	21.7	**18.3**	15.3	21.7
None	**22.6**	21.3	24.0	**21.9**	20.5	23.4	**20.6**	19.1	22.2	**17.8**	13.7	23.0

Regular e-cigarettes		Wave 8			Wave 9			CA Wave		
		Percentage	Lower 95% CI	Upper 95% CI	Percentage	Lower 95% CI	Upper 95% CI	Percentage	Lower 95% CI	Upper 95% CI
**Age group**										
	18-24	**3.3**	2.4	4.4	**4.3**	3.2	5.7	**3.9**	1.5	9.9
	25-44	**5.8**	5.2	6.6	**6.9**	6.1	7.8	**7.6**	5.7	10.1
	45-64	**4.9**	4.3	5.5	**5.2**	4.6	5.9	**4.1**	3.4	5.1
	65+	**1.9**	1.6	2.4	**2.1**	1.7	2.6	**2.3**	1.2	4.5
**Gender**										
	Male	**5.0**	4.5	5.6	**5.6**	5.1	6.3	**5.8**	4.4	7.4
	Female	**3.6**	3.3	4.0	**4.2**	3.7	4.7	**3.8**	2.9	4.9
**Ethnicity**										
	White	**4.5**	4.1	4.9	**5.0**	4.6	5.5	**4.8**	4.0	5.7
	Asian	**2.0**	1.4	3.0	**2.3**	1.5	3.5	**1.8**	0.7	4.3
	Black	**1.8**	1.0	3.5	**2.4**	1.3	4.7	**0.1**	0.0	0.7
	Mixed	**3.8**	2.2	6.7	**7.3**	3.8	13.6	**17.6**	4.6	48.8
	Other	**4.5**	1.6	11.8	**4.9**	1.7	13.3	**1.7**	0.2	12.1
**Education level**										
	Degree	**2.9**	2.5	3.4	**3.4**	2.9	3.9	**3.0**	2.4	3.8
	ALevel	**5.3**	4.1	6.7	**5.9**	4.7	7.5	**4.8**	3.1	7.4
	GCSE	**6.0**	5.3	6.8	**6.6**	5.8	7.5	**7.0**	5.1	9.5
	None	**4.7**	4.1	5.5	**5.3**	4.5	6.2	**5.4**	3.3	8.8
